# MaTSE: the gene expression time-series explorer

**DOI:** 10.1186/1471-2105-14-S19-S1

**Published:** 2013-11-12

**Authors:** Paul Craig, Alan Cannon, Robert Kukla, Jessie Kennedy

**Affiliations:** 1 Universidad Tecnológica de la Mixteca, Carretera a Acatlima Km. 2.5 Huajuapan de León, Oaxaca., C.P. 69000, México; 2 Edinburgh Napier University, School of Computing, Merchiston Campus, Edinburgh Napier University, 10 Colinton Road, Edinburgh, EH10 5DT, UK

**Keywords:** Information Visualization, Animation, Bioinformatics, Gene Expression, Time-course

## Abstract

**Background:**

High throughput gene expression time-course experiments provide a perspective on biological functioning recognized as having huge value for the diagnosis, treatment, and prevention of diseases. There are however significant challenges to properly exploiting this data due to its massive scale and complexity. In particular, existing techniques are found to be ill suited to finding patterns of changing activity over a limited interval of an experiments time frame. The Time-Series Explorer (TSE) was developed to overcome this limitation by allowing users to explore their data by controlling an animated scatter-plot view. MaTSE improves and extends TSE by allowing users to visualize data with missing values, cross reference multiple conditions, highlight gene groupings, and collaborate by sharing their findings.

**Results:**

MaTSE was developed using an iterative software development cycle that involved a high level of user feedback and evaluation. The resulting software combines a variety of visualization and interaction techniques which work together to allow biologists to explore their data and reveal temporal patterns of gene activity. These include a scatter-plot that can be animated to view different temporal intervals of the data, a multiple coordinated view framework to support the cross reference of multiple experimental conditions, a novel method for highlighting overlapping groups in the scatter-plot, and a pattern browser component that can be used with scatter-plot box queries to support cooperative visualization. A final evaluation demonstrated the tools effectiveness in allowing users to find unexpected temporal patterns and the benefits of functionality such as the overlay of gene groupings and the ability to store patterns.

**Conclusions:**

We have developed a new exploratory analysis tool, MaTSE, that allows users to find unexpected patterns of temporal activity in gene expression time-series data. Overall, the study acted well to demonstrate the benefits of an iterative software development life cycle and allowed us to investigate some visualization problems that are likely to be common in the field of bioinformatics. The subjects involved in the final evaluation were positive about the potential of MaTSE to help them find unexpected patterns in their data and characterized MaTSE as an exploratory tool valuable for hypothesis generation and the creation of new biological knowledge.

## Background

Recent years have seen an explosion in the rate at which biological data is generated and utilized. High-throughput technologies such as microarrays and RNA sequencing, that allow biologists to conduct experiments that measure the expression of tens of thousands of genes simultaneously, are becoming increasingly accessible and online data repositories continue to expand to give biologists unprecedented access to data recorded under an increasingly diverse set of experimental conditions [1]. It is widely recognized that these new sources of data have great potential to improve the diagnosis, treatment, and prevention of diseases [2,3]. However, the very scale and complexity of the data that gives it such potential can also make effective analysis problematic. If we consider a typical microarray data-set, we know that it can report gene expression for up to around 40,000 genes over 4 conditions and 20 time points [4,5]. So, in a single experiment we can have over three million data values. Simply storing or transforming these quantities of data can be problematic before we even begin to consider a way to present the data so that an analyst can extract valuable information.

A general methodology employed for the analysis of large scale gene expression data has been to use filtering and clustering to disregard less interesting parts of the data and generate a more 'manageable' data abstraction [6]. This allows the data to be visualized and an analyst to detect the general trends determined by the particular clustering algorithm employed. A disadvantage of this course of action is that it can lead to the loss of certain characteristic patterns such as changing activity over intervals of time (for example Figure 1 from [7]). Here, a rise then a fall in expression found over a particular interval could suggest that a group of genes are related to a particular biological process and that that process is associated with the experimental conditions. These types of pattern involve less of the data than the general trends found by clustering but nonetheless show great potential to generate biological insight [8,9].

**Figure 1:**
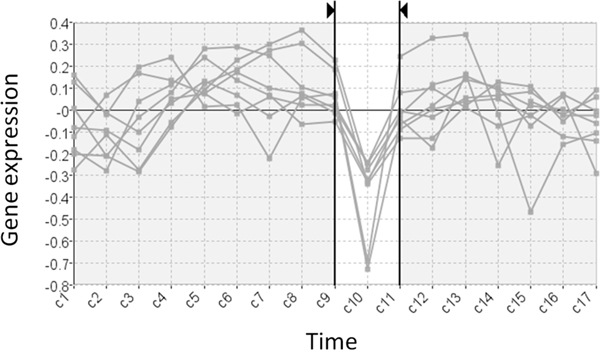
**A significant pattern occurring exclusively over an interval**. This filtered subset of genes share a pattern of falling activity from C9 to C10 and rising activity from C10 to C11.

In order to allow biologists to discover these types of patterns we developed the Time-series Explorer (TSE) technique [9-11]. This allowed us demonstrate the ability of an animated visualization technique to reveal previously unsuspected patterns of temporal activity in large scale microarray data. The gene expression time-series explorer (MaTSE) builds on this functionality to support a broader spectrum of user requirements for the analysis of gene expression time-series. This includes various improvements such as better performance, the accommodation of missing data, the facility to compare multiple groupings, a view of gene groupings and new functionality to support collaborative visualization. This paper describes the design and development of the new application.

### Related work

Current techniques developed for the exploratory analysis of large scale gene expression time-series data largely rely on procedures developed for the analysis of multidimensional data. These process the data to form clusters (groups) of genes based on the relative similarity of recorded expression (characteristic examples are [5,12,13]). Time-series data can be conceptualized as a specialized subset of multidimensional data [14] with the distinguishing characteristic that dimensions (time-points) are ordered. Clustering techniques do not account for this aspect of the data and, as a consequence, are ill-suited to revealing certain potentially significant patterns in the data [8]. Specifically, clustering tends to miss out patterns that occur exclusively over smaller intervals of an experiment's time frame such as previously described in Figure 1. Despite the fact that genes may share a similar profile over an interval of the data time-frame, differences in their expression over the remainder of the time course prevent them from being assigned to the same cluster. The application of feature extraction and fuzzy clustering could resolve this problem by accounting for the order of values and allowing genes to belong to more than one cluster [15]. This would, however, greatly increase the number of results by returning a cluster for every temporal pattern in the data without providing the user with any obvious way to explore the results.

Visual queries are an alternative method of analysis used to find genes with a specified pattern of activity over an interval of the data. To form a visual query the user draws a shape over a line-chart overview of the data to specify either: an acceptable range of values over an interval [16], a positive or negative change between time-points [16,17] or a profile of adjacent expression values [17]. Since line-chart overviews of time-series data are only really effective at showing the range of values at individual time points the biologists needs to already know the sort of expression pattern they are looking for before forming their query. So, while these methods allow a biologists find out which genes have a particular known expression pattern, they are incapable of allowing them to reveal any new unsuspected patterns.

When visual queries are combined with clustering views, the combination provides both an overview and a means of querying the data to look at interval patterns. Neither of these techniques is, however, capable of allowing a biologist to find *unsuspected *interval patterns and their combination does little to counter the limitation of either technique in this respect. Likewise, when different clustering views are linked they can be used to compare dominant trends but there is still no scope to find the patterns that each clustering view would be incapable of finding if applied in isolation. So, the interactive techniques that supplement clustering do *not *allow biologists to find interval patterns in their data.

Other techniques overlay line-chart views of gene expression data onto a gene network graph [18-22]. These techniques allow users to find interval patterns only if the genes involved already happen to be clustered together in the original gene network diagram. This makes it difficult to find co-expressed genes that do not already have some known association. These techniques are also limited by the amount of data that they can display and are generally used to view small parts of a gene network rather than provide an overview of the results of a high-throughput experiment.

### The Time-series Explorer

**Figure 2:**
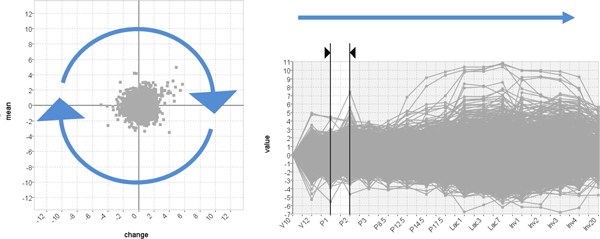
**The basic Time-series Explorer technique**. The technique uses tightly coupled line-chart and scatter-plot views of the data. The line-chart shows gene expression against time and allows the user to select an interval. The scatter-plot summarizes the data within the selected interval representing each gene as a single point. The y-axis represents mean activity and the x-axis change in activity. Adjusting the interval forward through time causes genes to move smoothly and predictably in an anticlockwise direction as expression changes from low to rising to high falling back to low etc.

The Time-series Explorer technique [9-11] (see Figure 2) was developed to overcome the limitation of existing techniques in order to allow biologists to explore large scale gene expression time-series data to find unsuspected patterns of temporal activity (such as the pattern shown in Figure 1). The technique employs two primary coordinated views of the data: a line-chart and a scatter-plot. The line-chart has two jobs. Firstly, it provides an overview of the data-set by overlaying value versus time representations of the recorded activity for all genes. The line-chart also allows the user to specify an interval of time. The scatter-plot summarizes the data within the selected interval by representing each gene as a single point. Genes are positioned so that their translation along the Y-axis corresponds to activity over the selected interval and translation along the X-axis corresponds to change-in-activity from the start to the end of the selected interval. As the line-chart view controls are adjusted and the selected interval is moved (with start and end times of the selected interval moved independently or in parallel), the positions of genes in the scatter-plot are recalculated with repeated continuous adjustments of the selected interval resulting in an animation. This allows users to perceive patterns of gene activity over time.

## Implementation

MaTSE [23] (additional file 1) builds on the functionality of TSE to support a broader spectrum of user requirements for the analysis of gene expression time-series. In order to ensure that the application would address a broad range of real world user requirements we held a one day workshop attended by biologists from diverse sub-disciplines such as Vascular Biology, Immunology, Bioinformatics, Statistics, Developmental Biology, Agriculture, Botany and Inflammation. This, and subsequent follow-up meetings, allowed us document the limitations of the Time-series Explorer and identify additional requirements for a new application. An example of a limitation was that patterns found were not measurable in TSE and that the biologists wanted results to be quantifiable so they could be stored during an exploration and shared or referenced at a later date. Examples of additional functionality requested were; the ability to cross-reference results with existing gene groupings, the representation of missing data, support for multiple experimental conditions, and rescaling to support different types of data. Performance also needed to be improved for the software to handle larger data sets.

This requirements analysis informed development of MaTSE. The initial stages of development focused on the core functionality of the data-model and animated scatter-plot. The objectives here were to improve performance and refine the layout to support measurable queries. We then worked to incorporate the display of missing values, the visualization of multiple conditions, the visualization of gene groupings and finally, the functionality required to support cooperative visualization. This plan focused on delivering working prototypes to involve users as early as possible in the software life-cycle. It was felt that the more we could engage potential users in evaluation the more effective our final application would be at addressing a wide variety of relevant end-user requirements.

From previous experience evaluating bioinformatics tools [24-26] we found that biologists evaluating a technique working with their own data, or at least data directly related to their own work, tended to be more motivated and generate better results. Biologists working with their own data also tended to find it easier to generate scenarios for evaluation since these can be based on their own objectives rather than simply imagined. They are also less likely to misunderstand the data and falsely interpret faults with the application. In general the process of evaluation was more natural when the biologists could envisage more familiar scenarios. This was crucial for the development of a system such as MaTSE where the results of the analysis would depend a lot on how the user interacted with the system. A potential drawback of test subjects working on their own data is that users might focus on data-set properties that are of particular interest to themselves rather than leading phenomena in the data under study. This could lead to the development of a tool that was specialized toward a particular biologist or a smaller group of biologists. While the tool we planned to develop was specialized to a degree in that it would focus on exploratory analysis of temporal patterns, we also wanted the tool to be useful across different sub-disciplines. Therefore, to mitigate the bias of any one particular biologist or group of biologists, efforts were made to validate evaluation findings with the larger group of biologists involved in our initial user requirements meeting. This was done before results were used to generate new user requirements. While this was costly due to time it took for new requirements to be vetted, we considered that the overall benefits of users working on their own data far outweighed the disadvantages.

### Data import

MaTSE supports two separate repository file formats (GDS soft [27], MAGE-TAB [28]) and an additional native CSV based file format for users who wish to import their data from a Microsoft excel or equivalent spreadsheet. Data import handles multi-condition data, replicates, ratios, and missing values. To ensure the data is properly interpreted a data import wizard helps users correctly identify data columns, compare conditions and re-order time points. The wizard also allows users to scale their data in order to better view the types of change they are most interested in. Scaling options include per value rescaling (log_2_, log_10_, cube root) and rescaling to a given data point or combination of data points (error weighted mean or median). Initially these options were only available through the import wizard, but evaluation showed that users often wanted to change rescaling options during an analysis session depending upon which option was most useful at any given point in time. Hence, rescaling options were eventually incorporated as an option that could be adjusted during analysis by selecting options in a toolbar directly above the main visualization panel.

### Scatter-plot view

**Figure 3:**
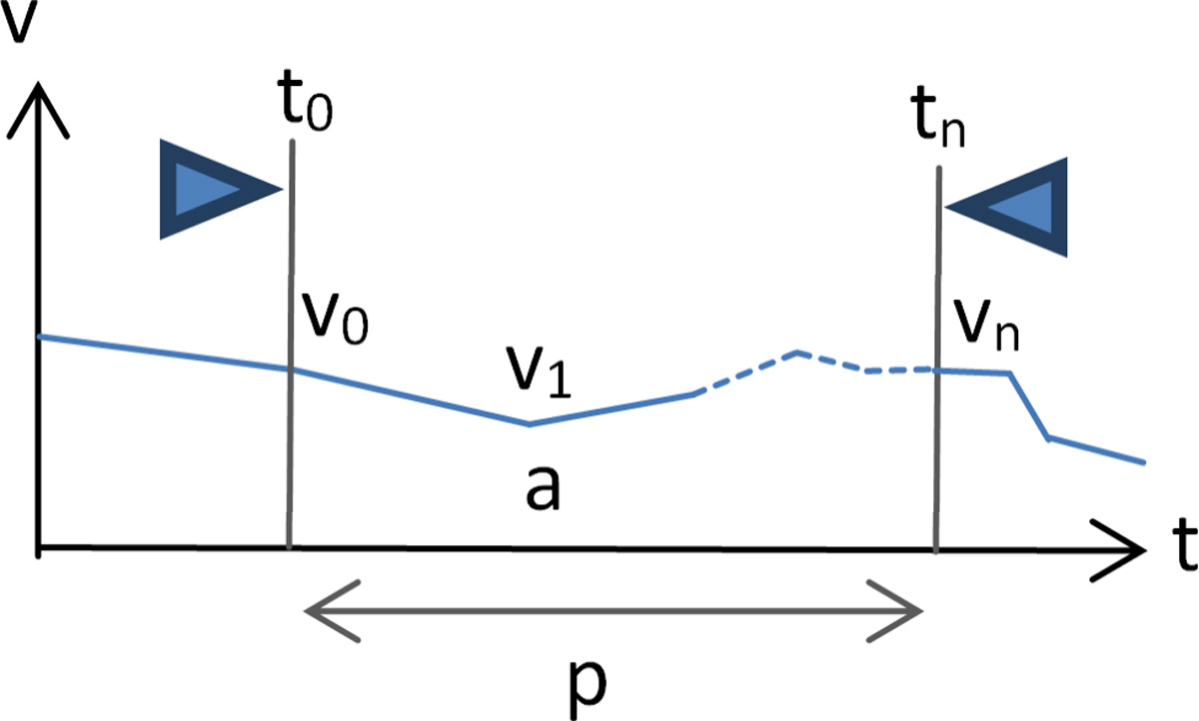
**Attribute definitions for Time-series Explorer and MaTSE scatter-plot layouts**. See table 1 for a comparison of the scatter-plot layouts in the Time-series Explorer and MaTSE.

The scatter-plot views of the Time-series Explorer application [11] and MaTSE [23] both summarize data inside a selected interval by representing each gene as a single point with gene's translation along the Y-axis corresponding to activity over the selected interval and translation along the X-axis corresponding to change-in-activity from the start to the end of the interval. The definitions of activity and change in activity, however, differ substantially (see Table 1 and Figure 3). For MaTSE, activity is calculated as the mean value over the interval and change in activity is measured as the difference or fold change between values. This allows MaTSE to overcome a significant limitation of TSE and operate with negative values and normalized data. MaTSE also avoids a limitation of TSE and allows the user to select a time interval with a duration of zero. Here, the × axis change-in-value is interpolated using the values for the nearest proper intervals directly before and after the selected interval. This interpolation is also used for animation between time-points. To avoid the interpolated values being used to form queries (which would lead to results being based on artefacts of the display rather than the data), the start and end of the interval selection automatically click to the nearest time-points for which expression is recorded at any time the user is not adjusting the interval selection.

**Table 1:** Comparison of the scatter-plot layouts in the Time-series Explorer and MaTSE.

		Time-series Explorer	MaTSE
**Pre-processing**		**None**	**None, log-rescaling and/or normalization**

**Limited to**		**Positive real numbers**	**Real numbers**

x - Axis	represents	Ratio	Difference or fold-change
	*p >*0	$\frac{v_{n}}{v_{0}}$	*v_n _− v*_0_
	*p *= 0	n/a	Interpolated value

y - Axis	represents	n/a	Mean value
	*p >*0	$\frac{a}{p}$	$\bar{v_{0\to n}}$
	*p *= 0	n/a	v

**Figure 4:**
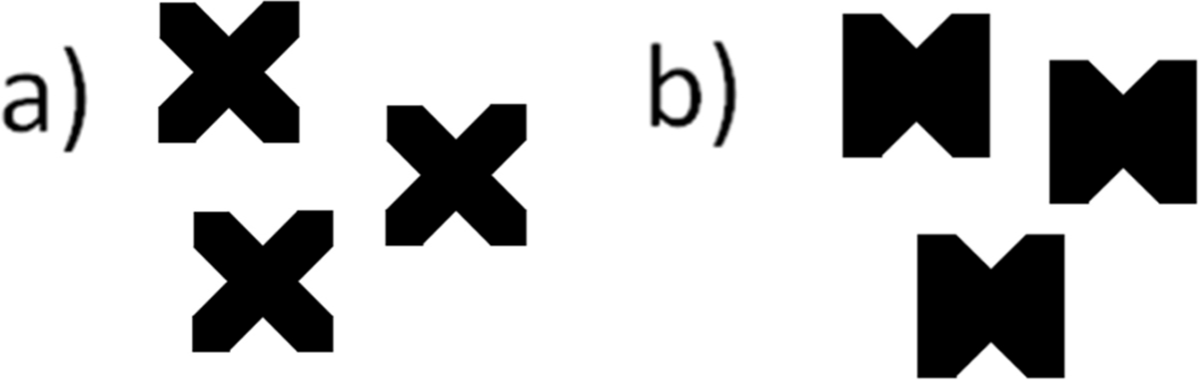
**Encoding for missing values in the scatter-plot**. a) Icon used when displacement along both axes is based on interpolated values. b) Icon used when only Y-axis displacement is based on interpolated values.

Attribute definitions are illustrated in Figure 4.

### Visualizing missing values

Gene expression data frequently suffers from missing values. This can be either due to experimental reasons or post-processing where the variability of recorded values makes it inappropriate to include any value in the resulting data-set. For the development of our gene expression time-series explorer application we decided against omitting the representation of missing values altogether since this might give the false impression that the genes for which data was not shown were not included in the experiment. Instead we decided to interpolate missing values where possible and change the manner in which they were represented in order to avoid them being attributed undue significance.

To represent missing or interpolated values, MaTSE uses dedicated visual attributes. These are dotted lines when interpolating over missing values in the line chart and dots with missing segments for interpolation in the scatter-plot (see Figure 4). This is consistent with visualization guidelines to use less 'ink' to represent data with less evidence. While other applications use annotation and animation for missing values [29], in MaTSE animation is already used to communicate the change in expression for genes and we have too many genes to annotate them individually without introducing excessive clutter.

### Visualizing multiple conditions

**Figure 5:**
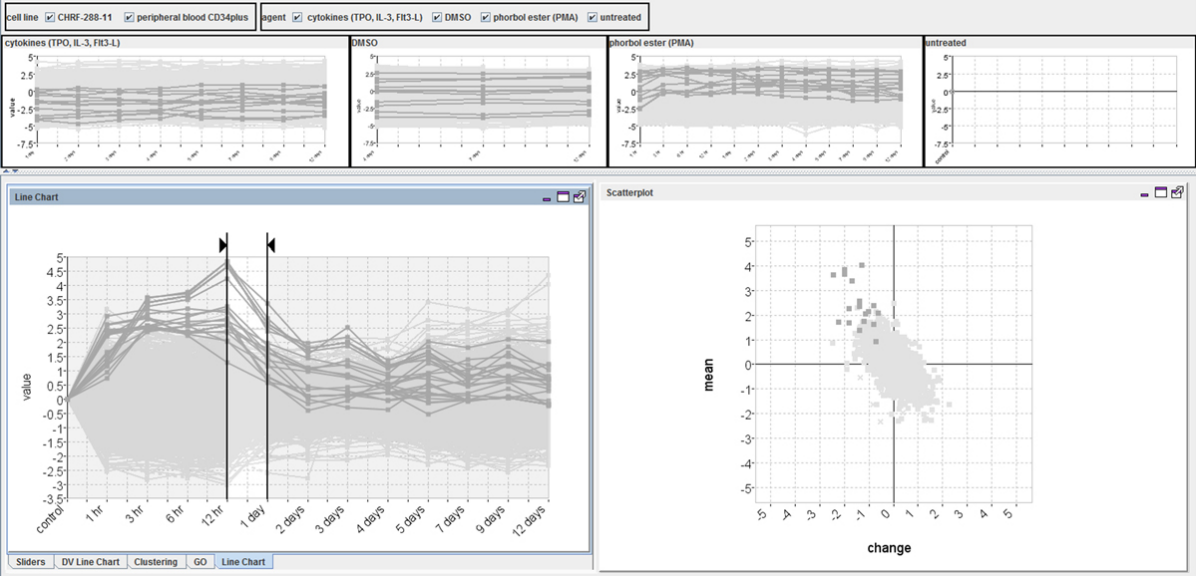
**Display of multiple conditions**. This uses line-chart overviews and a single linked scatter-plot and line-chart view for the current selected condition.

High throughput gene-expression experiments are often repeated with a single variable changed and biologists need to compare data generated under different conditions. Here the independent variable might be an environmental factor such as temperature, an exposure to a different treatment, or some form of genetic modification. The effect of these additional conditions on the data is to add another dimension and multiply the size accordingly. To support multiple conditions in MaTSE we provide the user with an overview line-chart for each condition and a linked scatter-plot and line-chart for a single in-focus condition (see Figure 5). This design allows users to compare patterns across conditions using line-chart views and explore to find patterns, or investigate patterns in more detail, as they would for a single-condition data-set using the linked line-chart and scatter-plot.

### Visualizing gene groupings

**Figure 6:**
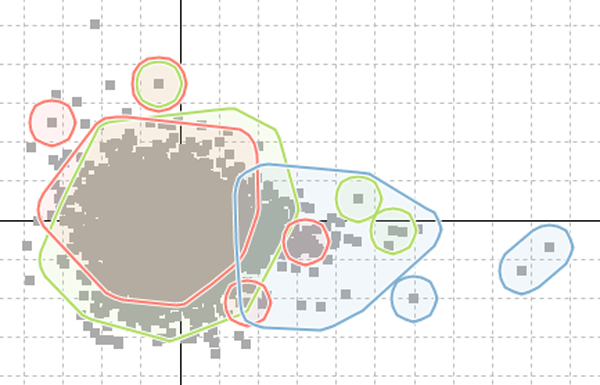
**Displaying gene groupings in the scatter-plot**. Smoothed outline shapes with transparent shading allow the effective visualization of up to four groups simultaneously.

In bioinformatics, genes groupings can indicate things such as functional similarity, the encoding of a common protein or co-expression under particular conditions. These groupings can be extremely useful for biologists when it comes to assessing to significance of any patterns found during the analysis of a data-set. In MaTSE, gene groupings can be imported from external sources, created from gene selections in the interface, visualized, and exported to files. The alternative methods for visualizing gene groupings are: colour coding, outline colour, symbols, areas with texture and colour, and smoothed outline shapes with transparent shading (see Figure 6). These employ the qualitative colour-coding schemes proposed by Brewer [30].

When we evaluated these different methods it was found that users considered outlined transparent shading to be the most effective and aesthetically pleasing option. While this method had a potential false-positive effect of circling genes that do not belong to the encoded group, users did not consider this to be a serious disadvantage. This was how they expected the highlighting to work and if they specifically wanted to see the individual genes of a grouping highlighted in the scatter-plot they could select those genes by clicking on the group name in the gene-grouping panel. In order to interpret the display the biologists were able to use the metaphor of a line drawn around a group of points on a projection of a scatter-plot over a whiteboard. This technique worked well with up to four individual gene-groups being viewed at the same time. The biologists involved in evaluation considered that this would be sufficient for the majority of their requirements.

### Cooperative visualization

**Figure 7:**
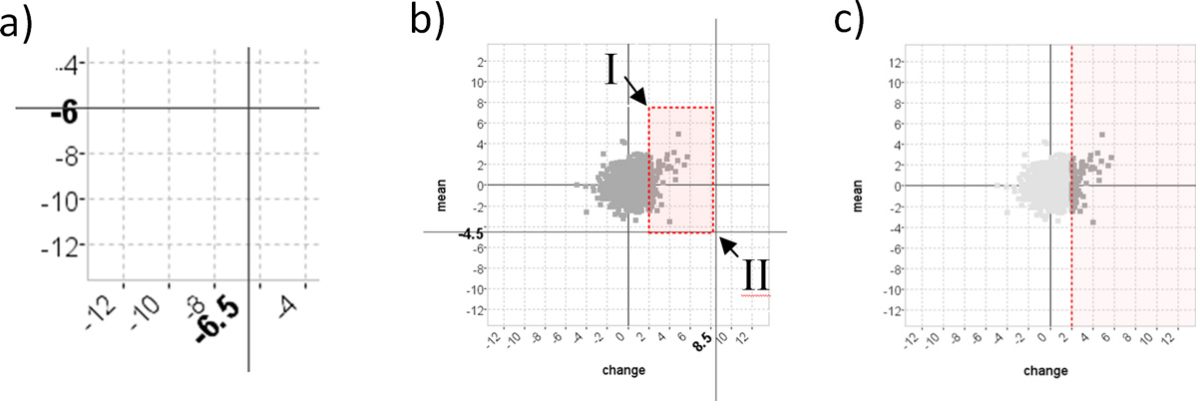
**Cooperative visualization in MaTSE**. a) Bold font labels on the axes describe the rounded-value cross-hair positions to inform the user before and during query specification. b) The user clicks on point I and drags to point II to form the box-query illustrated with dotted lines. c) The dotted line indicates the single threshold the user actually wants to set and the threshold sent to the MaTSE pattern browser as the recorded query.

An advantage of having scatter-plot coordinates based on measurable parameters in MaTSE is that it allowed us to adapt the interface to support a process of collaborative visualization where biologists are able to work together toward an understanding of the data and biological phenomena under investigation [31,32]. This process is based on entities called 'patterns' which are defined as findings or insights that can be summarized using a selection or group of selections together with their result. In MaTSE genes are selected by first selecting an interval then clicking and dragging to draw a box around groups of genes in the scatter-plot. Selections can be combined using Boolean logic AND, OR and NOT rules and the stored specification of a pattern includes the parameters used to form queries, the logic used to combine the queries and contextual data such as the data-set identifier and details of any transforms applied to the data during pre-processing. Patterns are automatically stored and listed in a 'patterns' panel [31]. This panel can also be used to annotate, restore, combine, refine or export patterns for them to be passed to other users. In order that patterns are understandable when they are recalled or shared, query parameters are restricted to rounded values (see Figure 7a) and superfluous parameters are removed as queries are formed (see Figures 7b and 7c).

## Results and discussion

**Figure 8:**
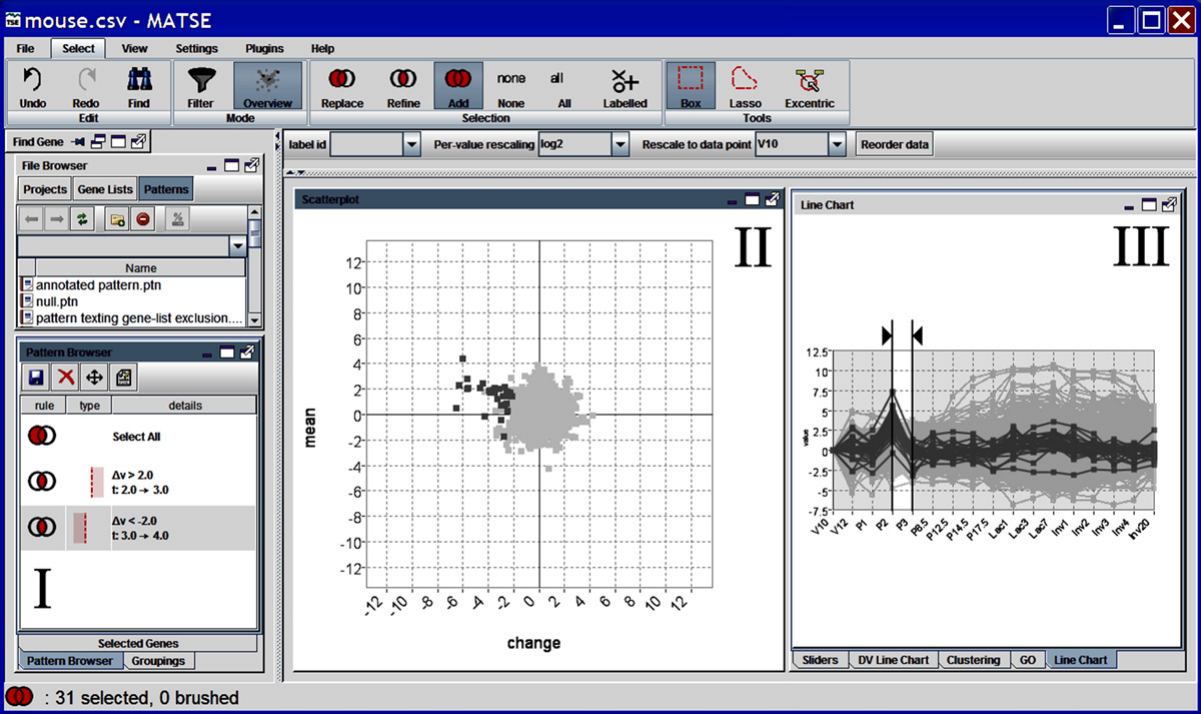
**A screenshot of the MaTSE interface**. Labeled components are I) the pattern-browser, II) scatter-plot and III) line-chart.

The final MaTSE prototype (see Figure 8) allows users to perform exploratory analysis of gene expression data by controlling linked line-chart and animated scatter-plot views to view patterns of gene activity over time and find unexpected patterns of changing activity. This can be achieved for with at least 50,000 genes with activity recorded over 12 time-points on a low spec desktop computer designed for personal use with 4GB RAM and a 2GHz CPU. MaTSE also allows the user to view missing data, visualize multiple conditions and overlay gene groupings. The user can also store the selections used to select genes. These selections are grouped together as 'patterns' which can be stored, restored, adjusted, exported and shared with other biologists (either by passing a file or uploading a pattern to the MaTSE web repository).

MaTSE provides a unique view of gene expression data that allows users to explore and find patterns of changing activity over intervals of time that have the potential to lead to biological insights [8,9] (for an example, see Figure 1). These patterns cannot be found using either clustering techniques or established techniques that allow the user to compose visual queries. Clustering tends to lose these patterns due to more dominant patterns over a larger time frame while visual queries rely on a pattern being suspected before the user composes a query.

MaTSE is also unique in that it allows queries to be adjusted in a predictable manner. While a number of tools allow users to share findings by saving and restoring application states (for example Spotfire DecisionSite [4] and Agilent Genespring), analysis steps cannot be adjusted in a predictable way or with adequate feedback of results. The majority of software applications used for the analysis of microarray data rely on clustering algorithms which prescribe a fixed set of gene clusters based on gene-gene activity similarity scores. A finding from this type of interface tends to be an individual cluster exhibiting a pattern of gene activity with a gene population that is correlated with a predefined gene grouping or biological pathway sourced from the literature. For a user to alter a query on which a clustering finding is based they would need to select an alternative grouping, specify alternative clustering parameters (such as the distance metric) or use an alternative clustering algorithm. In the former case the results would be unrelated to the original result and in the latter two cases the outcome is highly unpredictable as an entirely new set of gene clusters will be generated with little relation to the original set [33,34]. Visual query techniques also fail to provide adequate feedback of results when queries are adjusted since the overview provided is unable to reveal anything other than the range of values at individual time points [11]. Changes in activity are represented by angled lines between time-points in a line-chart and the majority of lines are occluded. Conversely, since MaTSE provides an overview of meaningful attributes for genes over the selected interval for every query that is formed (see Table 1), any specification or adjustment of query parameters has a predictable result with immediate feedback of results.

### Evaluation

Usability is a critical factor for the effective development of information visualization applications [35]. It was therefore important for the MaTSE project to employ a user-centred development methodology. To make use of user feedback throughout the software life-cycle we employed an iterative development strategy where a succession of prototypes would gradually introduce new functionality to be evaluated and refined before the production of a final prototype with full functionality. To ensure we considered the actual needs of a wide range of potential users, evaluation was conducted with real life representatives from all of our target user groups. These included academics, industrial users, bioinformaticians, experimental biologists, and users with a general knowledge of multiple areas.

During evaluation we used a mixture of techniques designed to elicit qualitative feedback from smaller groups of expert users [36]. These included interviews, demonstration and hands-on observation of real world exploration using the speak-aloud protocol and heuristic evaluations. Our final evaluation involved interviews and further real world testing in the form of case studies. To remove bias, these employed users not involved in evaluation sessions during the earlier stages of development cycle.

### Case studies

This section describes excerpts from two case studies used to demonstrate the functionality of MaTSE. The first of these studies was to compare results obtained with MaTSE with those obtained with other approaches. This involved repeating the process of finding a known pattern that was previously unexpected and found using TSE (originally documented in [28]) followed by analysis of the same data with alternative analysis techniques. The second case study involved biologists analyzing a data-set to test functionality such as the creation and visualization of gene groupings.

The first case study involved a data-set (contained in additional file 2) recording the expression of around 8,500 genes over 17 time points belonging to four successive stages of development in mouse breast tissue: virgin (days 10 and 12), pregnancy (days 1, 2, 3, 8.5, 12.5, 14.5 and 17.5), lactation (days 1, 3 and 7) and involution (days 1, 2, 3, 4 and 20). A more complete description of the data is made by Stein et al and full annotated data is available online [37]. This data-set was analyzed first using MaTSE then with software that allowed users to combine clustering and visual querying.

**Figure 9:**
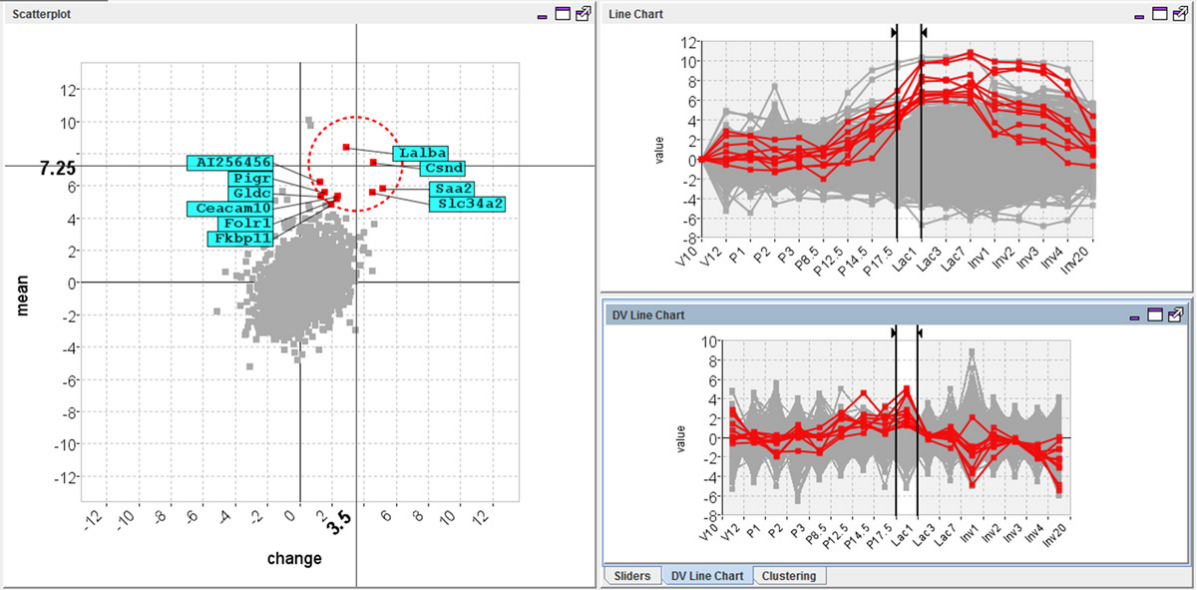
**Selecting genes known to have rising activity at the start of lactation**. The controls on the line-chart are adjusted to select the time interval from P17.5 to Lac 1 and genes with rising activity are highlighted in the scatter-plot using the excentric labelling tool.

Analysis with MaTSE began with the data being rescaled in the data import wizard so that values are expressed as the fold change from the first time point for each gene. This involved selecting a 'per-value rescaling' method of 'log_2_' and a 'data category to scale to' of 'V10'. The next stage of analysis involved finding a known pattern of activity to confirm that there were no problems with the data or experiment and that MaTSE displayed the data in the way the biologist expected it to. Here the biologist moved the line-chart view sliders to focus on the time period at the start of lactation (P17.5 to Lac1) and moved the mouse cursor over the scatter plot to look at genes with rising activity (see Figure 9). A number of the genes labelled were found to be familiar. This reassured the biologist that their understanding of the MaTSE scatter-plot layout was correct and that patterns found in the data could be trusted.

**Figure 10:**
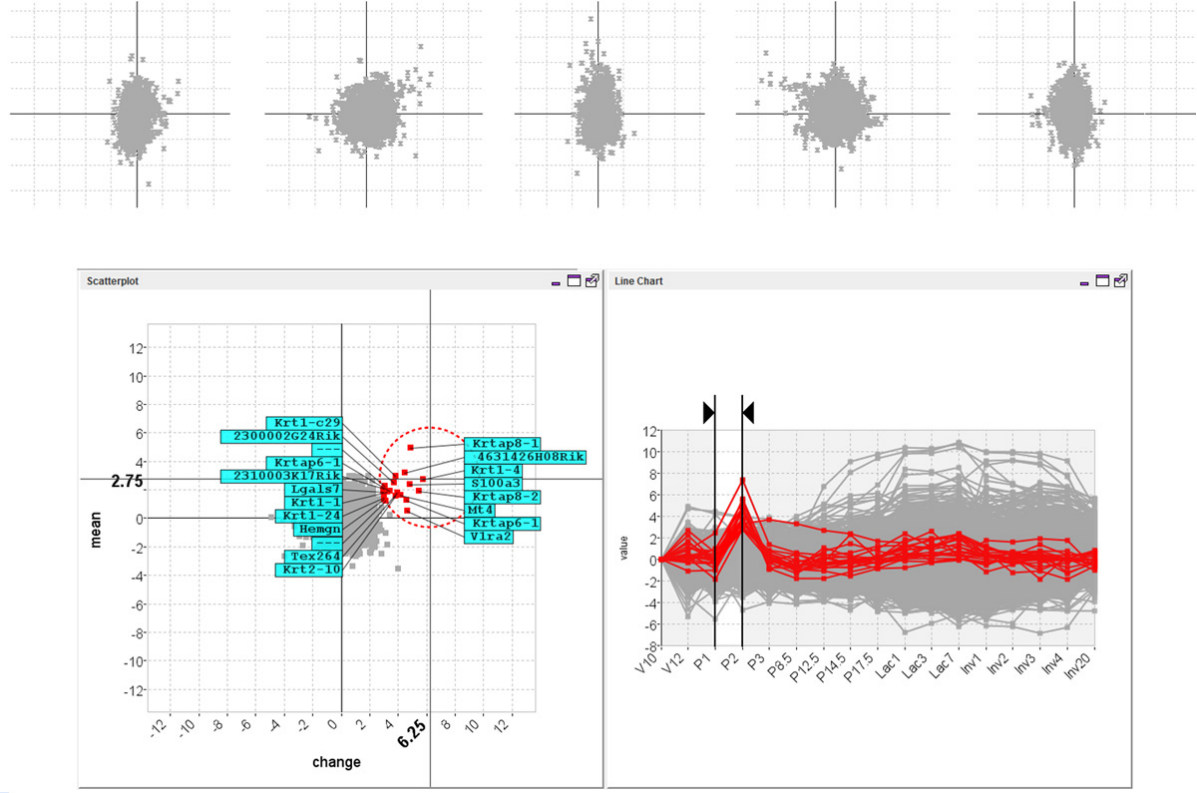
**Animating the MaTSE scatter-plot from interval P1 to P2 through to interval P2 to P3 reveals a group of outlying genes with rising then falling activity over a small interval of the time-course**. A small group of outlying genes were seen to swing first out to the right of the scatter-plot, then out to the left (top). This indicates that the genes share an outlying pattern of rising then falling activity over the time-frame of the animation. The effect in the actual animated scatter-plot is a lot stronger due to the Gestalt law of common fate which groups together objects with a common trend of motion. The pattern can be highlighted in the line-chart view when the genes are selected in the scatter-plot using the excentric labelling tool (bottom).

**Figure 11:**
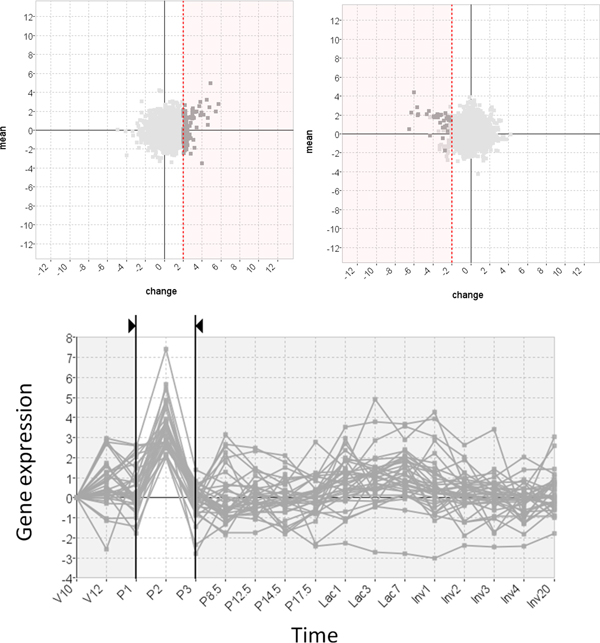
**Storing a pattern of temporal activity**. The pattern is stored by dragging a box around the genes in the scatter-plot (using the box tool from the Select menu) and shifting to another interval to refine the selection (top). The stored pattern can be seen as a selection in the line-chart view (bottom).

Next the biologist shifted their attention to finding more unsuspected patterns of activity. This involved selecting an interval fixed at one time-point and exploring the data by pressing 'rewind' then 'play' (from the View menu) to animate through the data-set and sporadically pausing to adjust the selected time interval manually. The resulting animation allowed them to find a number of general trends in the data such as a general flattening of the scatter-plot along the horizontal axes at the start of lactation. This indicated that there were a large number of genes with significant changes in their expression around this time and was a pattern that could be seen again when the data was clustered. The animation also allowed the biologist to find a previously unsuspected pattern of activity with potential biological significance. This was evident when the scatter-plot was animated from interval P1 to P2 through to interval P2 to P3 (see Figure 10 top). Here a small group of outlying genes were seen to swing first out to the right of the scatter-plot, then out to the left. This indicated that the genes shared an outlying pattern of rising then falling activity over the time-frame of the animation. The animation was stopped and the pattern was highlighted in the line-chart view by selecting the genes in the scatter-plot using an excentric labelling tool (Figure 10 bottom). The pattern was then stored by dragging a box around the genes in the scatter-plot (using the box tool from the Select menu) and shifting to another interval to refine the selection (Figure 11).

**Figure 12:**
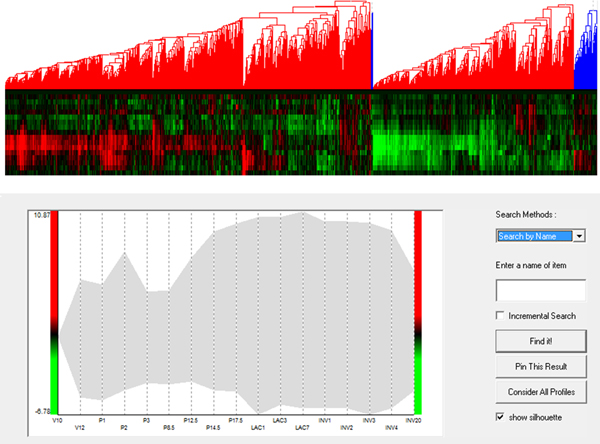
**Overview of the data in an application that supports both clustering and visual queries**. The heat-map dendrogram clustering view (top) shows the genes clustered using Average Link hierarchical clustering with Pearson's Correlation Coefficient as a distance measure. The visual query overview (bottom) uses a silhouette to illustrate the extreme values of gene activity over time. This type of application cannot be used to find interval patterns such as those shown in Figures 1 and 11.

**Figure 13:**
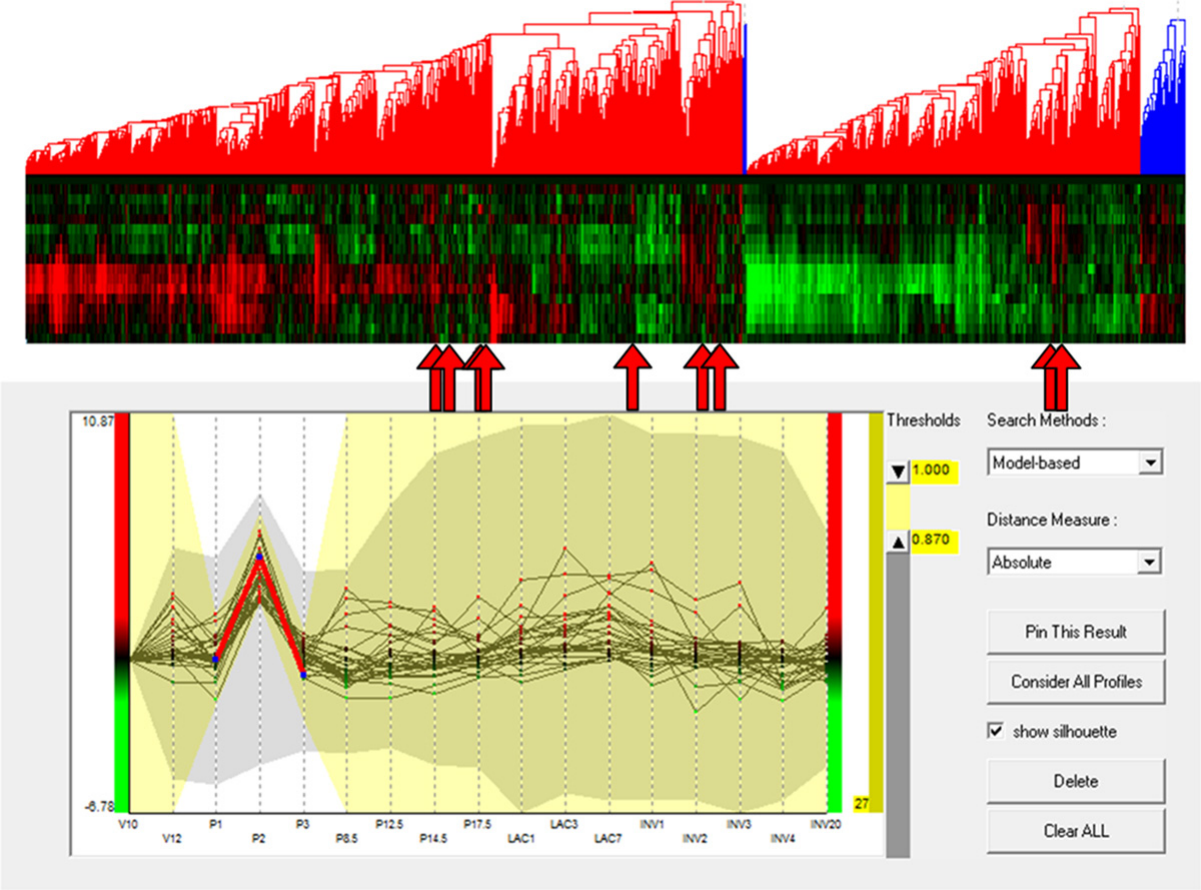
**A pattern of temporal activity selected in an application that supports both clustering and visual queries**. A known pattern of temporal activity has been selected in the visual query view (bottom) and with their positions highlighted using arrows in the cluster view (top). Despite the fact that the genes share a similar profile over an interval of the data time-frame, more dominant patterns of expression over the remainder of the time course cause the genes to be assigned to different clusters (this is not, however, the case for all genes since some genes are close enough to each other in the cluster view that the arrows showing their position overlap).

To evaluate the added value of MaTSE and see which patterns could be found using other techniques the analysis was repeated using applications combining various cluster and visual-query views of the data. Figure 12 shows an example of this with the data being analyzed in the Hierarchical Clustering Explorer application [38] with Average Link hierarchical clustering using Pearson's Correlation Coefficient as a distance measure. Here, and with various other clustering methods, the data tended to cluster into two principle groups with activity either rising or falling after lactation. Within these groups it was possible to identify a number of smaller subgroups that often indicated an earlier or later rise or fall in activity. These patterns were not unexpected but definitely held biological significance and could undoubtedly be described as some of the most significant patterns in the data. However, the interval pattern found during the analysis with MaTSE was also considered to have potential biological significance but it was not possible to detect this pattern with any of the clustering methods used. Despite the fact that the genes shared a similar profile over an interval of the data time-frame, it was always the case that more dominant patterns of expression over the remainder of the time course would cause the genes to be assigned to different clusters. This phenomenon can be observed by selecting the known pattern from Figure 11 in a visual query view and highlighting the results in a cluster view of the same data (see Figure 13). Here the relative displacement of genes demonstrates how differently they tend to be clustered. This result is unsurprising since the objective of clustering is to find the most dominant patterns in the data and genes are assigned to clusters accordingly. Even if genes could be assigned to more than one cluster and a cluster was produced for every temporal pattern, the number of potentially significant temporal patterns is likely to be massive and there would be no obvious way to explore the results to find patterns.

The interval pattern found using MaTSE was also impossible to find using a visual query approach. Here, the line-chart overview was found to be unable to reveal anything other than the range of values at individual time points and if a biologist has no knowledge of the timing or the genes that form a pattern they would need to execute an impractical number of speculative queries before the pattern could be revealed. This lead our test subject to conclude that the use of an interactive visualization technique such as MaTSE, that allows the user to explore the data by filtering and navigating the data in a predictable manner, would be necessary to find this type of pattern.

**Figure 14:**
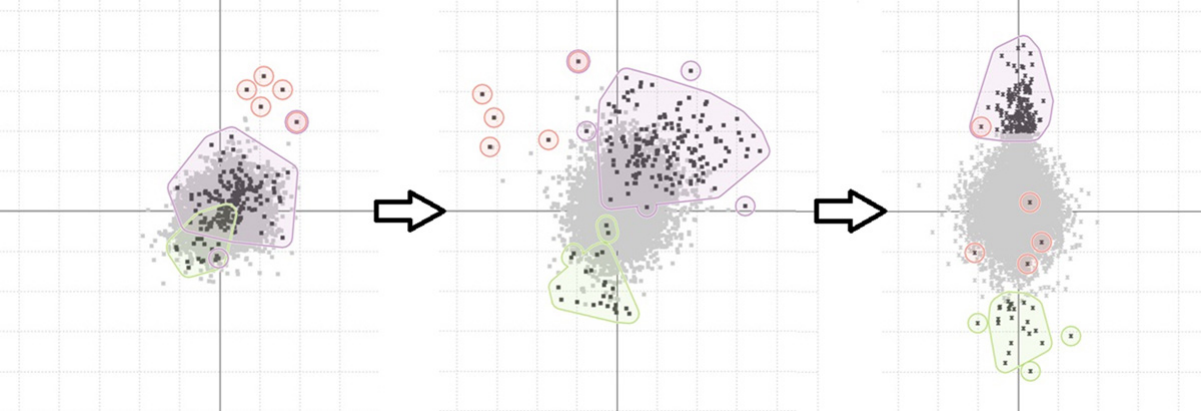
**Animating the scatter-plot to view patterns of activity among gene groupings**. Grouping outlines move as the scatter-plot is animated to reveal patterns of activity among the gene groupings.

Our second case study involved time course data (contained in additional file 2) examining osteoblast differentiation by comparing cells exposed to growth factors with control cells (series GSE3792 at NCBI GEO). This data records the activity of around 20,000 genes over 7 time points (day 4, 5, 6, 8, 16, 25 and 30). As with the previous data-set the data is rescaled so that values are expressed as the fold change from the first time point for each gene. During the analysis of this data-set, the user created 3 individual groups by selecting genes in the scatter-plot. These included an 'early rising' group of highly expressed genes at day 6-8, a 'downward' group of those genes with very low expression at Day 25-30; and an 'upward' group of those genes with highest expression at day 25-30). After selecting genes to create each group, the user scanned the names of the genes to check for known stem cell marker genes. This helped the user confirm that the groups were populated according to their expectations. After this the user selected a colour for each group (using the dropdown box next to the name of each grouping) and animated the scatter-plot across time (see Figure 14). This helped give the biologist a better impression of how the activity of the groups related to each other and allowed them to notice an overlap between the 'upward group' and the 'early risers' group from day 25 to 30. This was considered to be of potential biological significance and the name of the overlapping gene was noted for further investigation.

### Discussion

During the evaluation of MaTSE users generally expressed satisfaction and were positive about the potential of MaTSE to help find valuable patterns in their data. In general they believed that MaTSE would be of best use for exploratory analysis and hypothesis generation rather than for investigating known phenomena. It was suggested that a typical analysis workflow involving MaTSE would start with a more prescriptive method such as clustering to find the most significant patterns in the data. MaTSE could then be used to explore the data and find unsuspected patterns of temporal activity. This would be followed by statistical analysis of the results using a package such as R. It was also suggested that future work would benefit from fully harnessing the growing and highly valuable knowledge bases and annotation services such as the Gene Ontology [39], as well as supporting the use of the large sets of gene group lists built up by experienced biologists, which would make analysis more convenient for users.

Another suggestion from our test-users was that MaTSE could also be useful for the analysis of large scale time-series data other than that produced by microarray experiments. This might include the analysis of similar data obtained at the mRNA level, such as sequencing-based expression data [40], or time series proteome profiling data obtained at the protein level [41]. The analysis of these types of data is often similar to that of microarray data with biologists having the same general objective of finding groups of genes or proteins with certain common patterns of activity. MaTSE could also be used for non expression time-series data such as metabolite measurements [42] or multivariate environmental data. In general, the added value of MaTSE should be more pronounced for series with larger numbers of data elements and more time-points. Both these factors affect the number of temporal patterns in the data that would be lost if the data were only analyzed using clustering methods.

## Conclusions

In this paper we describe MaTSE, a new information visualization application for the analysis of gene-expression time-series data. MaTSE allows users reveal previously unsuspected patterns of gene activity over smaller intervals of an experiments time frame by allowing them to control an animated interval scatter-plot view of their data. MaTSE also supports multiple condition experiments, data with missing values and allows the display of up to four different gene groupings using a novel method using smoothed outline shapes with transparent shading. Users can also compose queries to define patterns that can be stored to be combined, refined, restored, annotated or exported for collaborative work. The utility of MaTSE is demonstrated in case studies showing how the final prototype can be used to find patterns, store patterns and explore gene-groupings.

The development of MaTSE demonstrates the benefits of an iterative software life cycle where developers work closely with potential users. It also allowed us to investigate some visualization problems that are likely to be common in the field of bioinformatics where large scale complex data is ubiquitous. Examples of these are how to highlight multiple groups in a densely populated scatter-plot and how to deal with superfluous parameters when recording a selection. In the final evaluation, our testers were positive about the potential of MaTSE to help them to find significant patterns in their data. Here MaTSE was characterized as an exploratory tool with potential for hypothesis generation and the creation of new biological knowledge. The biologists envisioned themselves using MaTSE as part of an iterative cycle of investigation together with a gene expression data repository and/or a database of gene groupings.

## Competing interests

The authors declare that they have no competing interests.

## Authors' contributions

Jessie Kennedy acted as principle investigator and project manager on the Scottish Enterprise proof of concept project from which most of this work is sourced. Paul Craig acted as chief developer on the project and developed the original Time-series Explorer application as part of his PhD supervised by Prof. Kennedy. Alan Cannon was responsible for evaluation and case studies while Robert Kukla developed the data model and web repository at http://www.matse.org.uk. All of the authors contributed to team meetings and discussions related to the design decisions described in the paper.

## Supplementary Material

Additional file 1 **MaTSE.jar**. Double click on this file and follow the instructions to install MaTSE. The software requires Java version minimum 1.7.0 to run. Click here for file

Additional file 2 **Data.zip**. Sample data files. Click here for file

## Declarations

This publication was funded by the IEEE Symposium on Biological Data Visualization (BioVis) as a supplement of highlights.

The articles in this supplement have undergone the journal's standard peer review process for supplements. The Supplement Editors declare that they have no competing interests.

This article has been published as part of *BMC Bioinformatics *Volume 14 Supplement 19, 2013: Highlights from the 2nd IEEE Symposium on Biological Data Visualization. The full contents of the supplement are available online at http://www.biomedcentral.com/bmcbioinformatics/supplements/14/S19.
